# Genetic spectrum and clinical characteristics of 3β-hydroxy-Δ^5^-C_27_-steroid oxidoreductase (HSD3B7) deficiency in China

**DOI:** 10.1186/s13023-021-02041-7

**Published:** 2021-10-09

**Authors:** Jing Zhao, Kenneth D. R. Setchell, Ying Gong, Yinghua Sun, Ping Zhang, James E. Heubi, Lingjuan Fang, Yi Lu, Xinbao Xie, Jingyu Gong, Jian-She Wang

**Affiliations:** 1 The Center for Pediatric Liver Diseases, Children’s Hospital of Fudan University, 399 Wanyuan Road, Minhang District, Shanghai, 201102 China; 2 Department of Pathology and Laboratory Medicine, Cincinnati Children’s Hospital Medical Center, Cincinnati, OH USA; 3 Department of Radiology, Children’s Hospital of Fudan University, Shanghai, China; 4 Department of Ultrasonography, Children’s Hospital of Fudan University, Shanghai, China; 5 Center for Molecular Medicine, Pediatrics Research Institute, Children’s Hospital of Fudan University, Shanghai, China; 6 Division of Gastroenterology, Hepatology and Nutrition, Cincinnati Children’s Hospital Medical Center, Cincinnati, OH USA; 7 Department of Pediatrics, Jinshan Hospital of Fudan University, Shanghai, China; 8 Shanghai Key Laboratory of Birth Defect, Shanghai, China

**Keywords:** Bile acid synthesis, Chenodeoxycholic acid, Genetic spectrum, *HSD3B7*, Renal lesions, 3β-hydroxy-Δ^5^-C_27_-steroid oxidoreductase deficiency

## Abstract

**Background:**

Biallelic variants in *HSD3B7* cause 3β-hydroxy-Δ^5^-C_27_-steroid oxidoreductase (HSD3B7) deficiency, a life-threatening but treatable liver disease. The goal of this study was to obtain detailed information on the correlation between the genotype and phenotype of HSD3B7 deficiency and to report on responses to primary bile acid therapy.

**Methods:**

The medical records of a cohort of 39 unrelated patients with genetically and biochemically confirmed HSD3B7 deficiency were examined to determine whether there exist genotype-phenotype relationships in this bile acid synthesis disorder.

**Results:**

In all, 34 of the 44 variants identified in *HSD3B7* were novel. A total of 32 patients presented early with neonatal cholestasis, and 7 presented after 1-year of age with liver failure (n = 1), liver cirrhosis (n = 3), cholestasis (n = 1), renal cysts and abnormal liver biochemistries (n = 1), and coagulopathy from vitamin K1 deficiency and abnormal liver biochemistries (n = 1). Renal lesions, including renal cysts, renal stones, calcium deposition and renal enlargement were observed in 10 of 35 patients. Thirty-three patients were treated with oral chenodeoxycholic acid (CDCA) resulting in normalization of liver biochemistries in 24, while 2 showed a significant clinical improvement, and 7 underwent liver transplantation or died. Remarkably, renal lesions in 6 patients resolved after CDCA treatment, or liver transplantation. There were no significant correlations between genotype and clinical outcomes.

**Conclusions:**

In what is the largest cohort of patients with HSD3B7 deficiency thus far studied, renal lesions were a notable clinical feature of HSD3B7 deficiency and these were resolved with suppression of atypical bile acids by oral CDCA administration.

**Supplementary Information:**

The online version contains supplementary material available at 10.1186/s13023-021-02041-7.

## Background

3β-hydroxy-Δ^5^-C_27_-steroid oxidoreductase (HSD3B7) deficiency is an autosomal recessive disorder of bile acid synthesis caused by biallelic pathogenic variants in the *HSD3B7* gene [1, 2]. As previously reported, patients may present with diverse clinical features, but mainly with neonatal cholestasis [3–5]. Some affected patients present with late-onset chronic liver disease or fat-soluble vitamin deficiency [4, 6, 7]. Definitive diagnosis of HSD3B7 deficiency is achieved by the detection of increased levels of atypical 3β-hydroxy-Δ^5^ bile acids in urine and confirmed by genetic analysis for variants in *HSD3B7 *[3]. Bile acid therapy with cholic acid (CA) or chenodeoxycholic acid (CDCA) has been shown to be effective and life-saving [8–10]. If untreated, HSD3B7 deficiency-associated liver disease may lead to liver failure requiring liver transplantation [9]. Comprehensive information on the clinical and genetic features of HSD3B7 deficiency is limited by the fact that worldwide there have been < 100 cases reported of this rare disorder and consequently there is a paucity of data on genotype-phenotype associations. [1, 2, 4, 7, 10–21]. Due to the lack of urinary analysis by mass spectrometry to establish the biochemic diagnosis in some regions of the world, the more frequent use of panel or whole exome sequencing has led to molecular analysis playing an increasing role in establishing an early diagnosis. However, interpreting clinical significance of genetic variants remains a critical roadblock [22, 23]. Underlying pathogenic variants are often classified as variants of uncertain significance (VUS) for lack of data, which could lead to under-recognition of this treatable disorder.

The aim of this study was to present the genetic spectrum, clinical features and treatment outcome of a large cohort of Chinese patients with a confirmed HSD3B7 deficiency, and discuss the possible impacts of *HSD3B7* variants on the clinical phenotype.

## Methods

### Patients

We retrospectively reviewed the findings from 39 patients who were diagnosed with HSD3B7 deficiency at Children’s Hospital of Fudan University between the years 2009–2020. This included five patients (P5, P9, P11, P13, and P14) that were reported previously [17–19, 24]. In 33 patients, the diagnosis was established by clinical features, serum liver biochemistries, urinary bile acid analysis by fast atom bombardment ionization mass spectrometry (FAB-MS), and molecular analysis. In 6 cases (P3, P6, P36 –P39) where urine was not available for analysis, the diagnosis was suspected based on clinical characteristics and serum liver biochemistries, and then confirmed by genetic studies with parental verification. The following information was collated from patient records: gender, geographical origin, age at disease onset, age at first visit to our hospital, clinical features, laboratory findings, radiological studies, genetic data, type and duration of therapies, and responses to treatment.

This study was approved by the Ethics Committees on Human Research of the Children’s Hospital of Fudan University.

### Genetic study

Before December 2015, all exons and adjacent introns of *HSD3B7* (RefSeq NM_025193.4) were Sanger sequenced as described previously [17]. After January 2016, panel sequencing and Sanger confirmation were performed [25]. Large fragment deletions were confirmed by quantitive polymerase chain reaction (qPCR). Variants were annotated for frequency in public databases (Genome Aggregation Database and Exome Aggregation Consortium) and predicted pathogenicity in PROVEAN (http://provean.jcvi.org), Polyphen-2 (http://genetics.bwh.harvard.edu/pph2/), MutationTaster (http://www.mutationtaster.org/), SIFT (http://sift.jcvi.org) and FATHMM (http://fathmm.biocompute.org.uk). Variants with minor allele frequency < 0.005 and predicted to be pathogenic by at least one of the five programs were considered as predicted pathogenic variants. The American College of Medical Genetics and Genomics (ACMG) guidelines was used for clinical sequence interpretation [26].

To explore the possible genotype-phenotype association, all variants were categorized into two classes. Frameshift, nonsense, classical splicing variants and large fragment deletions predicted to result in nonsense mRNA decay or protein truncation were defined as null variants. Other variants, including missense, non-classical splicing and non-frameshift small indel types, were defined as non-null variants.

### Urinary bile acid analysis

Urine samples were collected before any treatment with the primary bile acid, chenodeoxycholic acid (CDCA), and analyzed at the Cincinnati Children’s Hospital Medical Center using FAB-MS mass spectrometry [9]. In patients with a suspected bile acid synthesis disorder, treatment with UDCA was terminated 5–7 days prior to collection of urine samples. Diagnosis of a HSD3B7 deficiency was based on the finding of a lack of the normal primary bile acid conjugates and the presence of the pairs of ions at m/z 469/485 (sulfate conjugates) and m/z 526/542 (glyco-sulfate conjugates) representing the atypical 3β-hydroxy-Δ^5^-bile acids that are the signature metabolites for this bile acid synthesis disorder. FAB-MS analysis of urine was also used to monitor the therapeutic response to primary bile acid therapy [1, 27, 28].

### Management

After the confirmation of the diagnosis, CDCA (initially 4–10 mg/kg/d) was prescribed. Serum biochemistries were measured every week until the jaundice resolved and thereafter monthly until the normalization of liver function tests was achieved. Urinary bile acid analysis and renal ultrasound were repeated every 6 months. Dose adjustments of CDCA were based on the findings of reductions in the levels of atypical 3β-hydroxy-Δ^5^-bile acids from the urinary bile acid analyses combined with changes in the serum biochemistries, including serum transaminases and GGT.

### Statistical analysis

Statistical analysis was performed using SPSS 17. Mann-Whitney test, Fisher’s exact test and Spearman correlation were performed. Values for *p* < 0.05 was considered statistically significant.

## Results

### The genetic spectrum of HSD3B7 deficiency

There were 44 pathogenic/predicted pathogenic variants identified (Table 1, Additional file 2:  table S1 and S2). Of these, 23 were missense variants (42.3%), 5 were nonsense variants (16.7%), 3 were splice site variations (5.1%), 12 were small (< 15 bp) deletions or insertions (34.6%) and one was a 1.2-kb deletion (1.3%). Information regarding paternity and maternity revealed homozygotes in 14 patients (35.9%) and compound heterozygotes in 17 patients (43.6%). In eight patients (20.5%), parental verification was not performed (Table 1).

**Table 1:** *HSD3B7* variants in 39 patients with 3β-hydroxy-Δ^5^-C_27_-steroid oxidoreductase (HSD3B7) deficiency

Patients	Zygosity	Location	Nucleotide change (NM_025193.4)	Predicted amino acid change (NP_079469.2)	ACMG classification^†^		Parental derivation	Geographical origin
					Classification	Evidence		
P1	Hom	Ex6	c.1031 A > G	p.Tyr344Cys	LP	PS3 + PM2_S + PP4	Paternal/maternal	Zhejiang
P2	Het	Ex1	c.45_46delAG	p.Gly17Leufs*26	P	PVS1 + PS4 + PM2_S	Maternal	Jiangxi
	Het	Ex6	c.988_990delACC	p.Thr329del	LP	PM2_S + PM3 + PM4 + PP3	Paternal	Jiangxi
P3	Hom	Ex6	c.968 C > T	p.Thr323Met	VUS	PM2_S + PP3	Paternal/maternal	Jiangsu
P4	Het	Ex5	c.683G > A	p.Arg228Gln	LP	PS4 + PM2_S + PM3 + PP3	Paternal	Shandong
	Het	Ex6	c.1040delT	p.Leu347Argfs*70	LP	PVS1 + PM2-S	Maternal	Shandong
P5	Het	Ex1	c.45_46delAG	p.Gly17Leufs*26	P	PVS1 + PS4 + PM2_S	Maternal	Yunnan
	Het	Ex2	c.262G > C	p.Gly88Arg	VUS	PM2_S + PM3 + PP3	Paternal	Yunnan
P6	Hom	Ex4	c.484_485delinsCC	p.Ser162Pro	VUS	PM2_S + PM5 + PP3	Paternal/maternal	Jiangsu
P7	Hom	Ex5	c.544delC	p.Leu182Cysfs*4	LP	PVS1 + PM2_S	Paternal/maternal	Guizhou
P8	Hom	Ex4	c.474delC	p.Tyr159Ilefs*27	LP	PVS1 + PM2_S	Paternal/maternal	Jiangxi
P9	Het	Ex5	c.543dupG	p.Leu182Alafs*16	P	PVS1 + PS4_M + PM2_S + PM3	Maternal	Hebei
	Het	Ex6	c.790 C > A	p.Pro264Thr	VUS	PM2-S + PM3 + PP3	Paternal	Hebei
P10	Het	Ex6	c.781G > A	p.Asp261Asn	VUS	PM2_S + PM3 + PP3 + PP4	NA	Jiangxi
	Het	Ex6	c.1079G > A	p.Trp360Ter	LP	PVS1-Strong + PM2_S + PP4	NA	Jiangxi
P11	Het	Ex3	c.401G > A	p.Gly134Glu	VUS	PM2_S + PP3 + PP4	NA	Anhui
	Het	In4	c.532-3 C > G		VUS	PM2_S + PP4	NA	Anhui
P12	Het	Ex5	c.682 C > T	p.Arg228Trp	VUS	PM2-S + PM5 + PP3 + PP4	NA	Hebei
	Het	Ex6	c.1061G > C	p.Arg354Pro	VUS	PM2_S + PP3 + PP4	NA	Hebei
P13	Het	Ex4	c.503G > A	p.Trp168Ter	P	PVS1 + PS4 + PM2-S	Maternal	Hubei
	Het	Ex5	c.683G > A	p.Arg228Gln	LP	PS4 + PM2_S + PM3 + PP3	Paternal	Hubei
P14	Het	Ex1	c.147G > A	p.Trp49Ter	LP	PVS1 + PM2_S	NA	Sichuan
	Het	Ex4	c.503G > A	p.Trp168Ter	P	PVS1 + PS4 + PM2-S	NA	Sichuan
P15	Het	Ex4	c.503G > A	p.Trp168Ter	P	PVS1 + PS4 + PM2-S	Paternal	Xinjiang
	Het	Ex5	c.569G > A	p.Arg190His	VUS	PM2_S + PM3 + PP3 + PP4	Maternal	Xinjiang
P16	Hom	Ex5	c.682 C > T	p.Arg228Trp	VUS	PM2-S + PM5 + PP3 + PP4	Paternal/maternal	Jilin/Shandong
P17	Hom	Ex6	c.988_990delACC	p.Thr329del	LP	PM2_S + PM3 + PM4 + PP3	Paternal/maternal	Henan
P18	Het	Ex5	c.543dupG	p.Leu182Alafs*16	P	PVS1 + PS4_M + PM2_S + PM3	Maternal	Gansu
	Het	Ex5	c.683G > A	p.Arg228Gln	LP	PS4 + PM2_S + PM3 + PP3	Paternal	Gansu
P19	Het	Ex1	c.45_46delAG	p.Gly17Leufs*26	P	PVS1 + PS4 + PM2_S	NA	Sandong
	Het	Ex6	c.770 A > G	p.Tyr257Cys	VUS	PM2_S + PP3	NA	Sandong
P20	Het	Ex5	c.683G > A	p.Arg228Gln	LP	PS4 + PM2_S + PP3	NA	Guangxi
	Het	Ex5	c.683G > T	p.Arg228Leu	VUS	PM2_S + PM5 + PP3	NA	Guangxi
P21	Het	Ex5	c.561T > G	p.Cys187Trp	VUS	PM2_S + PP3 + PP4	NA	Hunan
	Het	Ex5	c.586G > A	p.Gly196Ser	VUS	PM2_S + PP3 + PP4	NA	Hunan
P22	Het	Ex3	c.346T > C	p.Cys116Arg	VUS	PM2-S + PM3 + PP3 + PP4	Paternal	Henan
	Het	Ex6	C.964_965dup	p.Leu324Argfs*94	LP	PVS1 + PM2_S + PP4	Maternal	Henan
P23	Hom	Ex4	c.503G > A	p.Trp168Ter	P	PVS1 + PS4 + PM2-S	Paternal/maternal	Sandong
P24	Het	Ex5	c.676 C > T	p.His226Tyr	VUS	PM2-S + PM3 + PP3 + PP4	Maternal	Shandong
	Het		c.-205_323-108del		P	PVS1 + PM2-S + PP4	Paternal	Shandong
P25	Het	Ex4	c.503G > A	p.Trp168Ter	P	PVS1 + PS4 + PM2-S	Maternal	Hubei
	Het	Ex6	c.743G > C	p.Arg248Pro	LP	PM2-S + PM3 + PM6 + PP4	Assumed de novo^‡^	Hubei
P26	Hom	In3	c.431 + 2dupT		LP	PVS1 + PM2-S	Paternal/maternal	Yunnan
P27	Hom	Ex4	c.485_487delGCA	p.Ser162del	VUS	PM2-S + PM4 + PP4	Paternal/maternal	Zhejiang
P28	Het	Ex5	c.683G > A	p.Arg228Gln	LP	PS4 + PM2_S + PP3	Paternal	Hunan
	Het	In5	c.694 + 2T > C		LP	PVS1 + PM2_S + PM3	Maternal	Hunan
P29	Het	Ex2	c.173_174del	p.Val58Glufs*14	LP	PVS1 + PM2_S	Paternal	Shandong
	Het	Ex3	c.371T > C	p.Leu124Pro	VUS	PM2-S + PM3 + PP3 + PP4	Maternal	Shandong
P30	Het	Ex5	c.557 C > T	p.Thr186Met	VUS	PM2-S + PP3	Maternal	Shandong
	Het	Ex6	c.968 C > G	p.Thr323Arg	VUS	PM2-S + PP3	Paternal	Shandong
P31	Het	Ex1	c.45_46delAG	p.Gly17Leufs*26	P	PVS1 + PS4 + PM2_S	Paternal	Henan
	Het	Ex5	c.543dupG	p.Leu182Alafs*16	P	PVS1 + PS4_M + PM2_S + PM3	Maternal	Henan
P32	Hom	Ex4	c.499G > A	p.Glu167Lys	VUS	PM2-S + PP3	Paternal/maternal	Jiangxi
P33	Het	Ex6	c.698 A > G	p.Asn233Ser	VUS	PM2_S + PM3 + PP3	Paternal	Shandong
	Het	Ex6	c.1033G > T	p.Glu345Ter	LP	PVS1 + PM2_S	Maternal	Shandong
P34	Het	Ex6	c.920_931delGGCTGCTGCGGC	p.Trp307_Pro311delinsSer	LP	PM2-S + PM3 + PM4 + PP4	NA	Shanxi
	Het	Ex5	c.543dupG	p.Leu182Alafs*16	P	PVS1 + PS4_M + PM2_S + PM3	NA	Shanxi
P35	Het	Ex1	c.45_46delAG	p.Gly17Leufs*26	P	PVS1 + PS4 + PM2_S	Paternal	Jiangsu
	Het	Ex2	c.319 C > T	p.Gln107Ter	LP	PVS1 + PM2_S	Maternal	Jiangsu
P36	Het	Ex1	c.45_46delAG	p.Gly17Leufs*26	P	PVS1 + PS4 + PM2_S	Paternal	Hunan
	Het	Ex6	c.905delA	p.Asn302Metfs*18	LP	PVS1 + PM2_S + PM3	Maternal	Hunan
P37	Het	Ex3	c.402_403insG	p.Pro135Alafs*2	LP	PVS1 + PM2_S + PM3	Maternal	Anhui
	Het	Ex4	c.503G > A	p.Trp168Ter	P	PVS1 + PS4 + PM2-S	Paternal	Anhui
P38	Hom	Ex5	c.543dupG	p.Leu182Alafs*16	P	PVS1 + PS4_M + PM2_S + PM3	Paternal and maternal	Shanxi
P39	Hom	Ex4	c.503G > A	p.Trp168Ter	P	PVS1 + PS4 + PM2-S	Paternal and maternal	Yunnan

*Het* heterozygous, *Hom* homozagous, *Ex* exon, *In *Intron, *P* pathogenic, *LP* likely pathogenic, *VUS* variant of uncertain significance; PVS, pathogenic very strong, *PS* pathogenic strong, *PM* pathogenic moderate, *PP* pathogenic supporting

^†^According to the American College of Medical Genetics and Genomics interpretation guidelines

^‡^Without confirmation of paternity and maternity

Among the 44 variants, 10 were reported previously in the literature and 34 were novel [16–19, 24, 29]. All 34 novel variants were absent or with very low frequency (less than 1/10,000) in Genome Aggregation Database and Exome Aggregation Consortium. All were predicted to cause deleterious disruptions to the protein by at least one of the five programs: PROVEAN, MutationTaster, PolyPhen-2, SIFT and FATHMM software (Additional file 2: Table S1). According to ACMG standards and guidelines, 1 out of 34 novel variants were assigned as a “pathogenic variant,” 14 as “likely pathogenic,” and the remaining 19 as “VUS” (Additional file 2: Table S1).

**Fig. 1:**
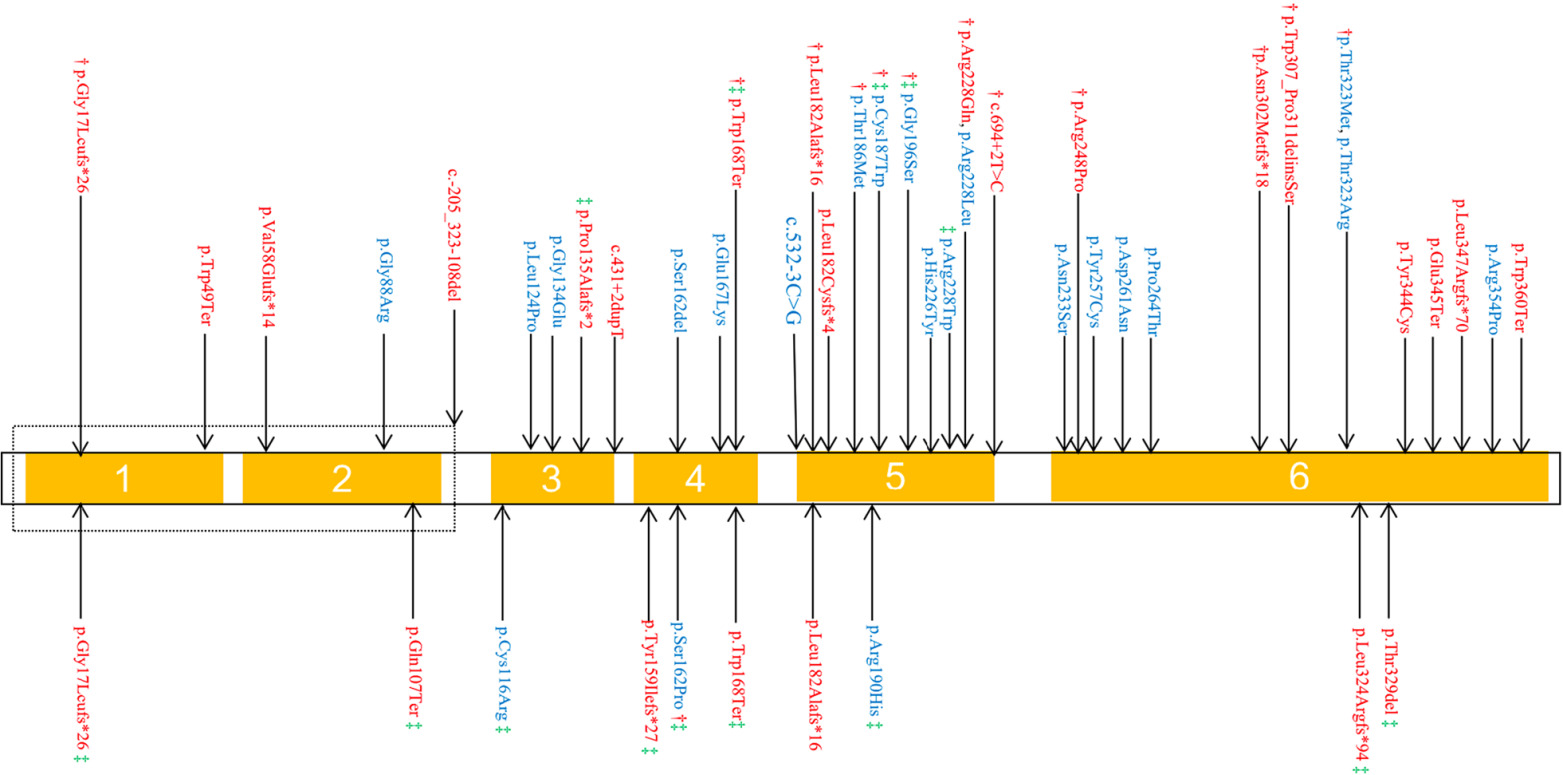
Schematic representation of variant locations in *HSD3B7* from 39 children with biochemically confirmed HSD3B7 deficiency. A total of 44 variants were identified. Each arrow represents one variant. Variants detected in patients presenting as neonatal cholestasis are shown on the top and variants detected in those patients with late onset occurrence of the disease are shown on the bottom. Variants from patients that underwent liver transplantation, or died are marked with †, and variants from patients with renal lesions are marked with ‡. Pathogenic or likely pathogenic variants are shown in red and variants with uncertain significance in blue

The variants identified were spread throughout the *HSD3B7* gene. Over 75% of patients carried an *HSD3B7* variant on exon 4, 5 or 6 (Fig. 1). The four most common variants were c.45_46delAG (n = 6, 7.7%) in exon 1, c.503G > A (n = 9, 11.5%) in exon 4, c.543dupG (n = 6, 7.7%) and c.683G > A (n = 5, 6.4%) in exon 5.

### Clinical data and laboratory evaluation

Among the 39 patients enrolled, 24 were male and 15 were female. Four patients (P2, P6, P24, P26) had one sibling respectively with neonatal cholestasis that died before 3 years of age. Table 2 summarizes the clinical features, liver biochemistries, urinary bile acid analysis, medical treatment, and outcome.

**Table 2:** Clinical features, urinary bile acid profiling, treatment, and outcome of patients with HSD3B7 deficiency

Patients	Gender	Age at onset	Age at first referral^†^	Presenting symptoms	Liver biochemistries		Urinary bile acids profiling^§^	Treatment after diagnosis	Status/age at last follow-up	Liver biochemistries	
					TB/DB (µmol/L)	ALT/AST (U/L)				TB/DB (µmol/L)	ALT/AST (U/L)
P1	M	1.5mo	5.7mo	Neonatal cholestasis, hepatomegaly	85.6/36.6	159/154	+	UDCA × 2 y, CDCA× 10y1mo	Normal/12y	5.6/2.4	32/22
P2	M	10d	16.5mo	Renal cysts, abnormal liver biochemistries, hepatomegaly with a history of transient neonatal cholestasis	24.7/20.1	128/72	+	UDCA × 2.5 y, CDCA ×7y6mo	Normal/11.2y	11.4/4	13.7/23
P3	M	5d	4.5mo	Neonatal cholestasis, hepatomegaly	133.9/65.5	36/85	NA	NA	Liver failure, then to lost follow-up/8mo	488.4/343.1	268/356
P4	F	7d	4.5mo	Neonatal cholestasis, hepatosplenomegaly	137.3/102	51/164	+	NA	Died/10mo	NA	NA
P5	M	5d	3.7mo	Neonatal cholestasis, hepatosplenomegaly	157.7/122.3	521/356	+	CDCA× 7y	Normal/7.3y	11.3/4.7	5/15
P6	M	16.8y	17.2y	Cholestasis, hepatosplenomegaly and then liver failure	96/68	62/46	NA	NA	Died/17.2y	720/593	179/104
P7	M	1mo	2.2mo	Neonatal cholestasis, coagulopathy, abdominal hematoma	123.9/75.7	157/132	+	NA	Lost follow-up/2.2mo	260.7/195.5	244/625
P8	F	3.5y	4.3y	Coagulopathy of vitamin K1 deficiency, abnormal liver biochemistries, hepatosplenomegaly	32/24	51/70	+	CDCA × 6y2mo	Normal/10.4y	13/2.6	25/9
P9	M	1mo	6.6mo	Neonatal cholestasis, hepatomegaly	151.3/108.75	812/819	+	CDCA × 5y8mo	Normal/6.2y	6.1/2.1	16.3/25.3
P10	M	2-3d	3.4mo	Neonatal cholestasis, hepatosplenomegaly	77.4/55.1	71/76	+	CDCA × 6y	Normal/6.3y	6/2.6	10.6/27.3
P11	M	2d	5.2mo	Neonatal cholestasis, hepatomegaly	164.1/109.9	376/297	+	CDCA × 12d	Liver biochemistries worsen/6mo	163.4/134.5	340/370
P12	F	1.5mo	2.6mo	Neonatal cholestasis	191.4/123.1	152/210	+	CDCA × 2y4mo	Normal/2.5y	10.6/2.4	16/31
P13	F	10d	2mo	Neonatal cholestasis, hepatomegaly	103.3/85.9	284/216	+	CDCA × 3y10mo	Normal/4y	12.4/3.7	16.8/31.6
P14	M	2mo	6.3mo	Neonatal cholestasis	335.9/236.8	768/608	+	CDCA × 4y	Normal/4.5y	16.6/2.14	13.2/24
P15	F	3d	6.6y	Recurrent cholestasis, splenomegaly	46.2/14.3	26/34	+	CDCA × 2y11mo	Normal/9.5y	11.5/2.4	20/25
P16	F	3d	5.8mo	Neonatal cholestasis	98/59.3	181/276	+	CDCA × 3y4mo	Normal/3.8y	5.1/1.9	13.9/30.2
P17	F	2mo	4.8mo	Neonatal cholestasis, hepatomegaly	81.9/37.7	75/197	+	CDCA × 2y5mo	Normal/2.8y	9.7/3.2	25.21/40.32
P18	F	1mo	4.6mo	Neonatal cholestasis	82.5/51.1	83/97	+	CDCA × 2y9mo	Normal/3.2y	7.6/2.8	17.48/26.43
P19	M	3d	1.7mo	Neonatal cholestasis	214.7/151	212/282	+	CDCA × 1y9mo	Normal/1.9y	3.1/1.7	37/31
P20	M	2d	5.5mo	Neonatal cholestasis, hepatosplenomegaly	138.1/68.8	327/485	+	CDCA × 2y5mo	Normal/2.8y	7/2	22/38
P21	M	10d	11.5mo	Neonatal cholestasis, liver failure, hepatosplenomegaly, pneumonia	309/213.6	72/154	+	CDCA × 10d, then liver transplanted	Aliver/4.8y	14.2/4.8	42.5/48.3
P22	M	3-4d	4.9y	liver cirrhosis, hepatosplenomegaly with a history of transient neonatal cholestasis	20.2/13.8	47/61	+	CDCA × 3y	Normal/7.9y	5/1.9	24.53/26.98
P23	M	1mo	8.7mo	Neonatal cholestasis	41.4/23.3	291/204	+	UDCA × 9mo	Hyperbilirubinemia resolved and transaminase slightly elevated /10mo	19.7/10.1	151/86
P24	M	11d	2.4mo	Neonatal cholestasis, hepatosplenomegaly	141.2/70.1	134/131	+	CDCA × 2y1mo	Normal/2.3y	7.9/3	31.4/43.2
P25	M	3d	3mo	Neonatal cholestasis, hepatosplenomegaly	204.9/101.3	279/393	+	CDCA × 3mo, then liver transplanted	Aliver/3.4y	327.2/150.2	116/289
P26	M	1mo	2.2mo	Neonatal cholestasis	88.9/49.5	107/137	+	CDCA × 3y2mo	Normal/3.4y	5/0.8	31/54
P27	M	7d	2.2mo	Neonatal cholestasis	125/85	40/132	+	CDCA × 1y1mo	Normal/1.3y	15.2/5.2	41.2/44.1
P28	M	18d	8mo	Neonatal cholestasis	165/59	46/294	+	CDCA × 3mo, then liver transplanted	Died /11mo	201.3/62	182/662
P29	M	3d	4.6mo	Neonatal cholestasis	96/37	111/167	+	CDCA × 2y2mo	Normal/2.5y	6.3/1.5	16/28
P30	M	7d	7.8mo	Neonatal cholestasis	128.6/69.3	84/406	+	CDCA × 3mo, then liver transplanted	Alive/2.7y	126.9/68.6	377/518
P31	F	4y	5.2y	Liver cirrhosis, splenomegaly	15.3/3.6	40/NA	+	CDCA × 2y6mo	Normal/7.7y	22.8/8.3	25/33
P32	F	3d	3.3mo	Neonatal cholestasis	170.4/93.9	290/153	+	CDCA ×1y5mo	Normal/1.8y	8.4/5	31/45
P33	F	3d	5mo	Neonatal cholestasis	74.6/42.8	100/200	+	CDCA × 12mo	Normal/1.4y	5.5/1.1	31/49
P34	M	3d	1.8mo	Neonatal cholestasis	141.2/92.1	119.8/136.7	+	CDCA × 3mo, then liver transplanted	Alive/1.3y	333.5/273	585.1/668.1
P35	F	4.5y	4.7y	Liver cirrhosis, splenomegaly	29.4/17.9	37.6/50.2	+	CDCA × 11mo	Hypersplenism improved/5.7y	11.8/4.8	16/24.9
P36	M	1mo	4.4mo	Neonatal cholestasis	436/327.1	938.4/1526.8	NA	CDCA × 1mo	Died /6mo	863.1/508.8	284.5/321.7
P37	M	1mo	1.8y	Neonatal cholestasis, liver failure	45/35.2	217.2/385	NA	CDCA × 12mo	Normal /2.4y	9.1/4.1	23.78/38.21
P38	F	3d	4mo	Neonatal cholestasis	88/66	189.3/170.5	NA	CDCA × 4mo	Hyperbilirubinemia resolved and transaminase slightly elevated /1.1y	8.2/3.1	67.06/62.44
P39	F	2d	4.7mo	Neonatal cholestasis	186.8/155.6	265/428.8	NA	CDCA × 2.5mo	Died/7mo	494.5/291.5	551/559
Reference range					3.4–17.1/ 0–6	9–50/15–40				3.4–17.1/ 0–6	9–50/15–40

+ positive, - negative; M, male; F, female; d, day; mo, month; y, year; NA, not available; UDCA, ursodeoxycholic acid; CDCA, chenodeoxycholic acid; † age at first visit to our center; ‡If renal imagine indicate renal lesions, the result is positive; §If FAB-MS profile show an absence or a lack of the normal primary bile acid conjugates and marked elevations of atypical 3β-hydroxy-Δ^5^-bile acids, the result is positive and supports a diagnosis of 3β-HSD deficiency; TB, total bilirubin; DB, direct bilirubin; ALT, alanine transaminase; AST, aspartate transaminase;

The median age of onset of symptoms was 10 days (range 2 days–16.8 years old). The median age at diagnosis was 4.8 months (range 1.7 months–17.2 years old). Depending on the onset age, we classified our patients into two groups. The first group included 32 patients presenting with neonatal cholestasis. The second group included 7 patients presented with a broad spectrum of symptoms after one year of age, including adolescence-onset cholestasis and liver failure (P6), liver cirrhosis with (P22) or without (P31, P35) a history of transient neonatal cholestasis, recurrent cholestasis (P15), renal cysts and abnormal liver biochemistries with transient neonatal cholestais (P2), and coagulopathy of vitamin K1 deficiency and abnormal liver biochemistries (P8).

Neonatal cholestasis with low serum GGT and serum total bile acids (sTBA), the latter measured by immunoassay, is a common feature of HSD3B7 deficiency. The serum GGT levels in the patients who were referred before one year of age ranged 8–70U/L and the range of the sTBA concentration was 0.2–85.4µmol/L. The concentration of sTBA was between 10 and 30 µmol/L in eight patients, five who had stopped UDCA treatment for five days, and > 30µmol/L in three patients of whom two (P4 and P38) were on UDCA therapy and one (P21) was in liver failure. These high sTBA would be expected in these three patients.

**Fig. 2:**
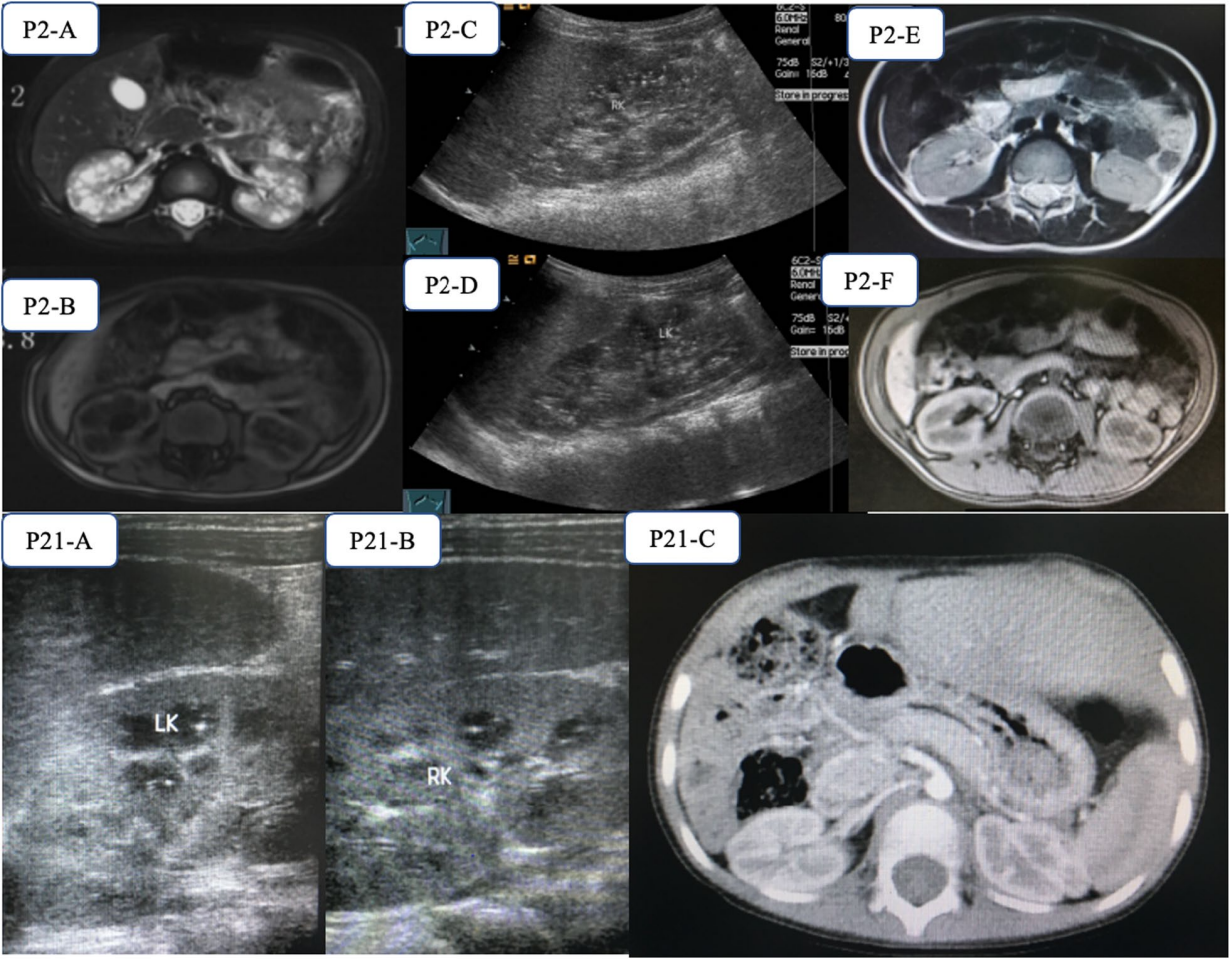
Evolution of renal lesions. In patient P2: before the commencement of chenodeoxycholic acid (CDCA) administration, diminished corticomedullary differentiation and multiple small cystic were revealed with high signal on T2WI-FS (P2-A) and low signal on T1WI-Flash (P2-B) in bilateral renal medulla by MRI. This presented as bilateral renal sponge-like degeneration with point-like deposition of calcium salts seen by renal ultrasound (P2-C and P2-2D). After CDCA treatment for a period of about 33 months (2012.2–2014.11), MRI showed disappearance of the renal lesions in this patient (P2-E and P2-F). In patient P21: calcium deposition in both kidneys was noticed by ultrasound (P21-A and P21-B) at first referral, and normal kidneys were shown by CT scan 18mo after liver transplantation (P21-C)

Renal images were collected from 35 patients before treatment with CDCA, of whom 10 (28.6%) had renal lesions, including renal cysts (n = 6), renal stones (n = 2), calcium deposition (n = 2 ), renal enlargement (n = 1) and multiple abnormal echoes in the calyx (n = 1) (Table 3; Fig. 2, Additional file 1: figure S1 and Additional file 1: S2). In these patients, the serum creatinine levels and urinalysis were all within the normal range. The patients with renal lesions (median age 3.1 years, range 3.7months to 17.2 years) were referred significantly later in age than patients that did not have identifiable renal lesions (median age 4.5 months, range 1.7 months to 5.2 years, *P* < 0.001).

**Table 3:** Manifestations of renal lesion and its revolution in patients with HSD3B7 deficiency

Patient	Age at first imaging	Renal imagine before chenodeoxycholic acid (CDCA) administration		Renal Tests		Management	Status of renal lesions/age at last follow-up
		Ultrasound	Magnetic Resonance Imagine	Serum Cr (µmol/L)	Urinalysis		
P2	16.5mo	Medullary sponge kidney with calcification	Multiple small cystic high signal in bilateral renal medulla	33.8	Normal	UDCA × 2.5 y, CDCA 10 mg/kg/day ×7y6mo	Normalized/11.2y
P5	3.7mo	Multiple abnormal echoes in the calyx	NA	13	Normal	CDCA 10 mg/kg/day × 7y	Normalized/7.3y
P6	17.2mo	Renal stones	Renal cysts	29	Normal	-	NA
P8	4.3y	Renal stones	NA	18	Normal	CDCA 10 mg/kg/day × 6y2mo	Normalized/10.4y
P15	6.6y	Renal cysts with calcification	NA	36	Normal	CDCA 8 mg/kg/day × 3mo, 10 mg/kg/day × 2y8mo	NA
P16	5.8mo	Renal cysts	Progressively abnormal signals	14	Normal	CDCA 10 mg/kg/day × 3y4mo	Normalized/3.8y
P21	11.5mo	Calcium deposition	NA	8	Normal	CDCA 8 mg/kg/day × 7d, 6 mg/kg/day × 4d, then liver transplanted × 18mo	Normalized/4.8y
P22	4.9y	Renal cysts	NA	25	Normal	CDCA 8 mg/kg/day × 21d, 5 mg/kg/day × 4mo, 6 mg/kg/day × 31mo	Normalized/7.9y
P35	4.7y	Bilateral renal enlargement	NA	29	Normal	CDCA 3 mg/kg/day × 11mo	Improved/5.7y
P37	1.8y	Renal cysts	Renal cysts	17	Normal	CDCA 4.5 kg/kg/day × 4mo	NA

*NA* not available

## Urinary bile acid analysis

**Fig. 3:**
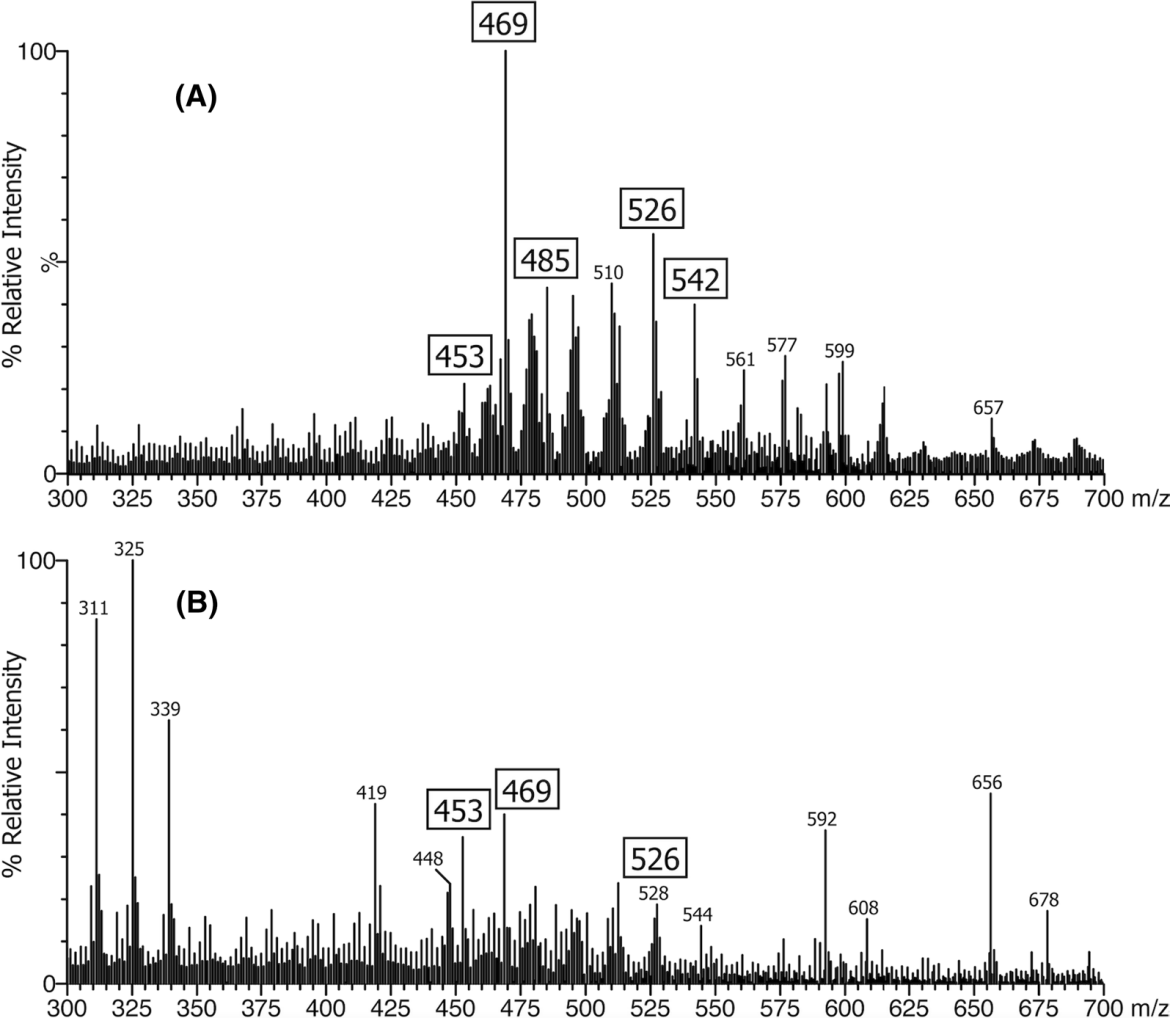
The negative ion FAB-MS spectrum of the urine for: **A** a patient with HSD3B7 deficiency revealing marked elevations in sulfate and glyco-sulfate conjugates of dihydroxy- and trihydroxy-cholenoic acids (i.e. unsaturated C24 bile acids) evidenced by the pairs of ions at *m/z* 469, 485 (sulfate conjugates) and *m/z* 526, 542 (glyco-sulfate conjugates) and **B** the mass spectrum of the urine from patient 21 which shows low intensity ions for these atypical 3β-hydroxy-Δ^5^ bile acid that are the biomarkers for HSD3B7 deficiency due to the more advanced liver disease and loss of synthetic function

Urine samples from 33 patients were collected and analyzed using FAB-MS. The profiles of 32 patients showed an absence or a lack of the normal primary bile acid conjugates and marked elevations in sulfate and glyco-sulfate conjugates of dihydroxy- and trihydroxy-cholenoic acids (ions at *m/z* 469, 485, sulfate conjugates; *m/z* 526, 542, glyco-sulfate conjugates) that are the biomarkers for the HSD3B7 deficiency. Compared with typical bile acid metabolities, the profile of Patient 21,who was in liver failure, showed only traces of these ion features, presumably because of significant loss of quantitative synthetic function (Fig. 3).

### Clinical follow-up and outcome

Apart from 2 patients (P4 and P6) that died before a diagnosis of HSD3B7 deficiency was established, 1 patient (P23) that refused oral CDCA therapy and 3 patients (P3, P7 and P11) that were lost to follow-up, 33 patients were treated with CDCA (initial dose ranging 3-10 mg/kg/d) and regularly monitored. The median follow-up peroid was 26mo (range 10 days to 10 + years). Of these, 24 (73%) achieved a complete normalization of serum liver biochemistries, 2 (6%) showed significant clinical improvement, 5 (15%) underwent liver transplantation, and 2 (6%) died. There was no significant difference in the age at diagnosis between the patient group consisting of the one that had a liver transplant and the deceased cases combined (median 4.9 mo, n = 7, range 1.8mo–11.5 mo) the group comprising the native liver survivors (median 4.8 mo, n = 26, range 1.4 mo–6.6 y, *P* = 0.874).

Of the 10 patients with renal lesions, one (P6) died before a definite diagnosis of HSD3B7 deficiency was made, two other patients (P15, P35) have yet to undergo repeat renal imaging. Renal ultrasonography was repeated in the other seven patients: Six patients were on continuous CDCA therapy, and one underwent a liver transplant (P21) 10 days after initiating bile acid therapy. Renal lesions eventually disappeared in all of these patients after a median duration of 13 mo (range 4mo to 36mo) and concomitant with a decrease or disappearance of atypical bile acids in urine and normalization of serum liver biochemistries (Fig. 2), save patient P37. In patient P37, renal ultrasound revealed bilateral renal enlargement improved after 11 months of CDCA treatment (left 87.9 mm*27.6 mm*24.3 mm, right 83.1 mm*31.6*37.6 mm, compared 105 mm*25.1 mm*29.7 mm and 89.8 mm*29.1*32.5 mm, respectively).

### Genotype-phenotype relationship

Genotypically, 12 patients were classified as harboring biallelic null variants, 15 patients as one null and one non-null variants, and 11 patients as biallelic non-null variants. Phenotypically, 32 patients were classified as neonatal cholestasis onset, 7 with childhood onset. The clinical outcome were classified as excellent for 27 patients (native liver suiviviors), and poor outcome for 12 (either died or were transplanted). No significant differences were observed in terms of age of disease onset or clinical outcome among the patients with different genotypes (Table 4). Similarly, there was no significant differences among patients with novel variants and other known variants (Additional file 2: table S4).

**Table 4:** Correlation of genotype and phenotype in patients with HSD3B7 deficiency

	Biallelic null variants (n = 12)	Single null variant (n = 15)	Biallelic non-null variants (n = 12)	Total (39)	Analysis (Spearman correlation)
*Group by onset age*					
Neonatal cholestasis	9 (75%)	12 (80%)	11 (92%)	32 (82%)	rs = 0.170, *p* = 0.300
Childhood onset	3 (25%)	3 (20%)	1 (8%)	7 (18%)	
*Clinical outcome*					
Native liver survivors	8 (67%)	12 (80%)	7 (58%)	27 (69%)	rs=-0.071, *p* = 0.668
Liver transplanted or death	4 (33%)	3 (20%)	5 (42%)	12 (30%)	

## Discussion

This study, the first of its kind, details the genotypic and phenotypic features of the largest collection of patients with HSD3B7 deficiency reported to date. Genetic analysis revealed 34 novel pathogenic or predicted pathogenic variants in the *HSD3B7* gene. Furthermore, our observation that 10 patients had renal lesions, and remarkably, treatment with oral CDCA or liver transplantation resolved these lesions concomitant with a suppression of the atypical 3β-hydroxy-Δ^5^-bile acids biomarkers, highlights renal lesions as an important clinical feature of this bile acid synthesis disorder.

We have described 34 novel variants in our patients; 19 novel variants were assigned as VUS, including 17 missense variants, 1 non-classical splice site variant and 1 non-frameshift (3 bp) deletion, which were absent or with very low frequency in public databases and were predicted pathogenicity by at least one of the five programs used. The diagnosis of these subjects was based on not only genetic analysis, but also on definitive features of the urinary bile acid profile, combined with the clinical fetaures and liver biochemistries. The bile acid profiles of 14 patients with 17 variants assigned as VUS were consistent with HSD3B7 deficiency which is important information for the pathogenicity assessment of these variants if they are detected in future patients. In two patients with the remaining two variants of uncertain significance (c.968 C > T and c.484_485delinsCC), serum TBA concentrations (measured by enzyme immunoassay) were low (< 10µmol/L) and consistent with expectations for a bile acid synthesis disorder [20]. Elevated atypical urinary bile acids and low serum TBA (measured when off UDCA therapy) enabled us to make the final diagnosis and to prove that these 19 novel variants of uncertain significance are likely pathogenic.

During the study peroid, 5086 patients with neonatal cholestasis were referred to our center. In our HSD3B7 deficiency patients, 32 presented as neonatal cholestasis. It is likely that HSD3B7 deficiency acounts for 0.6% of neonatal cholestasis in our single liver center. This would be consistent with the previously reported incidence of all bile acid synthesis diorders accounting for about 2% of unexplained cholestasis cases, with the HSD3B7 deficiency being the most common of the disorders [9]. A consistent finding was that liver biochemistries, revealed elevated serum conjugated hyperbilirubinemia, and transaminases, but normal GGT, consistent with previously reported cases [16]. Care is required when interpreting a routine serum TBA level obtained when the patient is receiving UDCA therapy because an elevated or slightly elevated serum TBA may not necessarily exclude a diagnosis of HSD3B7 deficiency in neonates. Although most patients with HSD3B7 deficiency showed good compliance to CDCA therapy, there were seven patients that did not respond to therapy, presumed to be due to the intrinsic hepatotoxicity of CDCA. For the patient P39, liver function indices worsened after contracting pneumonia and the patient later died at 7 months of age. Thus, infection might be another reason for the poor prognosis of some patients.

Our findings show that renal lesions in the face of normal renal chemistries have a prevalence of 28.6% in HSD3B7 deficiency and the most common renal involvement was renal cysts (5/10). Renal cysts have been described in a few patients but a causal association has not been previously confirmed [30]. In patients with HSD3B7 deficiency, primary bile acids are not synthesized and instead there is an accumulation of hepatotoxic 3β-hydroxy-Δ^5^-bile acids that leads to cholestasis that often progresses to subsequent liver failure. Urinary excretion consequently becomes the major route of elimination of these atypical bile acids. The cause of renal lesions is unclear but animal studies suggest that high concentrations of bile acids can be toxic to renal tubules and may generate or initiate renal lesions [31]. Whether chronic exposure of the kidney to high concentrations of the atypical 3β-hydroxy-Δ^5^-bile acids associated with HSD3B7 deficiency can explain the renal disease is conjecture. Significant was our finding that renal lesions appeared mainly in the older children and that these resolved upon suppression of bile acid synthesis, or after liver transplantation, both of which eliminate the production of 3β-hydroxy-Δ^5^-bile acids. No common variant was associated with renal lesions of HSD3B7 deficiency. These findings suggest that it may be the accumulation over time of 3β-hydroxy-Δ^5^-bile acids that appear to underlie the renal pathology.

In conclusion, this study presents a comprehensive description of the the *HSD3B7* genetic spectrum and clinical characteristics of HDS3B7 deficiency in a large cohort of infants and children from China. It concludes that the genotype is not a good predictor of the phenotype, or the clinical outcome. Furthermore, our data highlight the significant prevalence of renal lesions in HSD3B7 deficiency and that these lesions can be resolved by primary bile acid therapy. Thus, targeted renal evaluation, including serum biochemistries, renal ultrasound, and urinalysis, should be included in the standard work-up of children with HSD3B7 deficiency.

## Supplementary Information

**Additional file 1**. Renal images in additional patients.

**Additional file 2**. **Table S1**. Pathogenicity prediction of novel variants in *HSD3B7*;** Table S2**. Previously reported variants in *HSD3B7*;** Table S3**. Serum liver biochemistries at first referral and at last follow-up;** Table S4**. Correlation of genotype and phenotype in patients with HSD3B7 deficiency.

## Data Availability

The data sets generated during and/or analysed during the current study are available from the corresponding author on reasonable request.. All data generated and analyzed during this study are included in this article and its supplementary tables.

## Abbreviations

HSD3B7: 3β-hydroxy-Δ^5^-C_27_-steroid oxidoreductase
CA: Cholic acid
CDCA: Chenodeoxycholic acid
VUS: Variants of uncetain significance
FAB-MS: Fast atom bombardment ionization mass spectrometry
qPCR: Quantitive polymerase chain reaction
ACMG: The American College of Medical Genetics and Genomics
UDCA: Ursodeoxycholic acid
GGT: Gamma-glutamyl transpeptidase
TCH: Total cholesterol
TBA: Total bile acids
