# Supplementary material for: Genetic spectrum and clinical characteristics of 3β-hydroxy-Δ5-C27-steroid oxidoreductase (HSD3B7) deficiency in China
Source: Orphanet J Rare Dis. 2021 Oct 9;16:417. doi: 10.1186/s13023-021-02041-7 (PMC8501698; doi:10.1186/s13023-021-02041-7)

Additional file 1: figure S1. Renal lesions in three patients by abdominal ultrasound. P5-A and P5-B from the patient 5, P8-A and P8-B from patient 8, P15-A and P15-B from patient 15


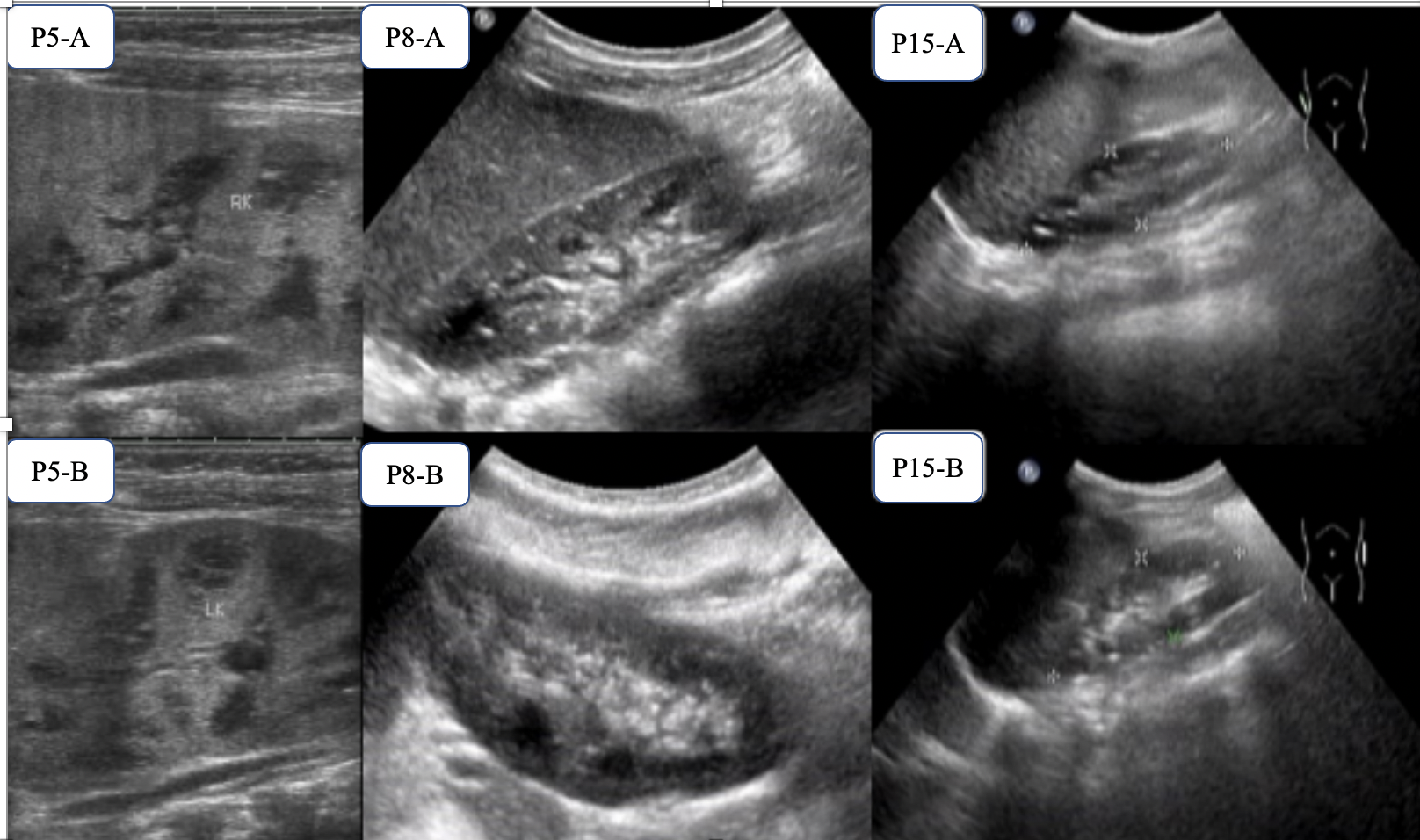


Additional file 1: figure S2. Renal lesions in patient 16 by MRI. P16-A and P16-B at the age of 4.5mo, P16-C and P16-D at the age of 5.5mo.


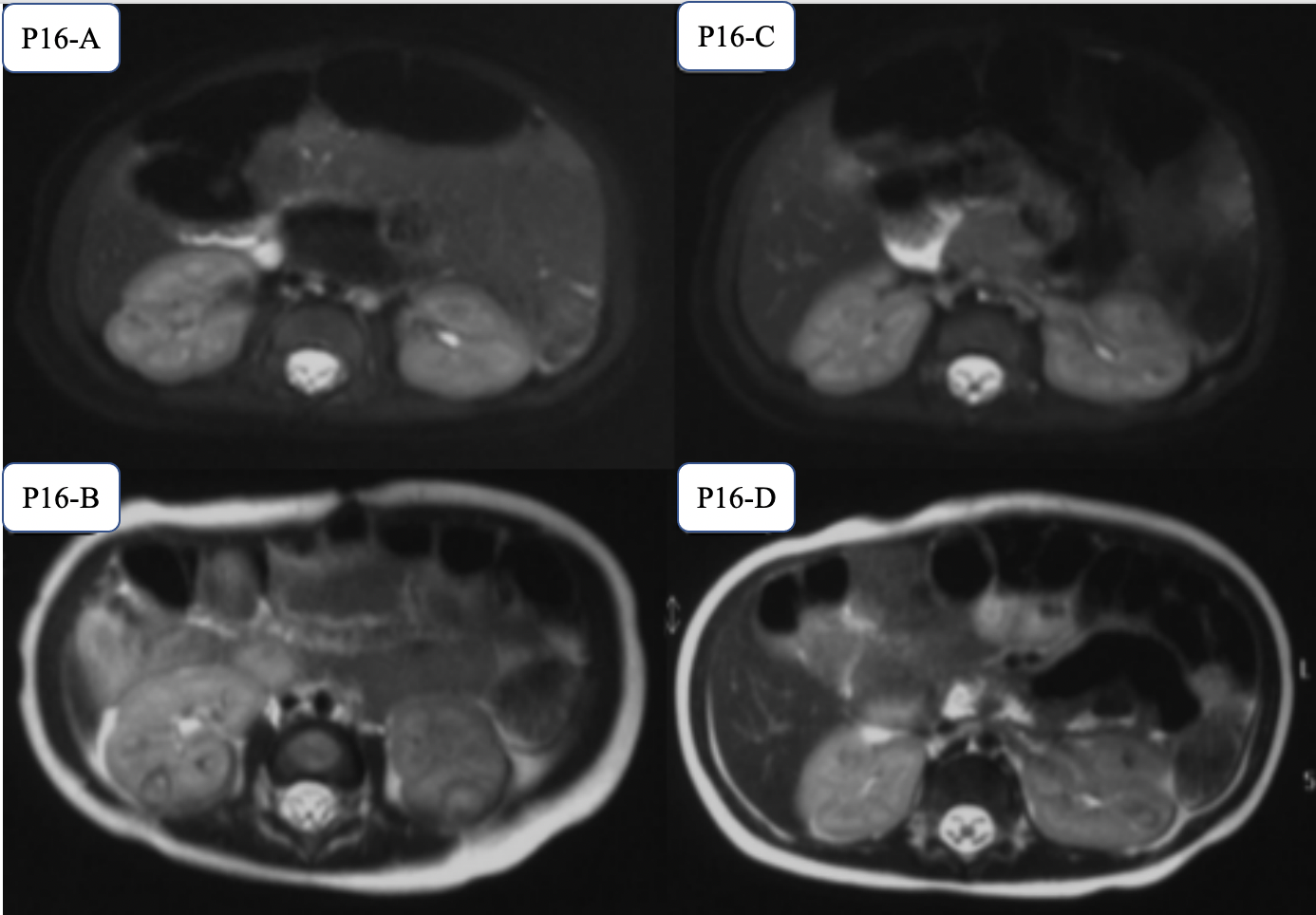

Supplement: Supplementary file 1 — Additional file 1. Renal images in additional patients. [file 13023_2021_2041_MOESM1_ESM.docx]
