# Supplementary material for: Genetic spectrum and clinical characteristics of 3β-hydroxy-Δ5-C27-steroid oxidoreductase (HSD3B7) deficiency in China
Source: Orphanet J Rare Dis. 2021 Oct 9;16:417. doi: 10.1186/s13023-021-02041-7 (PMC8501698; doi:10.1186/s13023-021-02041-7)
Supplement: Supplementary file 2 — Additional file 2. Table S1. Pathogenicity prediction of novel variants in HSD3B7; Table S2. Previously reported variants in HSD3B7; Table S3. Serum liver biochemistries at first referral and at last follow-up; Table S4. Correlation of genotype and phenotype in patients with HSD3B7 deficiency. [file 13023_2021_2041_MOESM2_ESM.docx]

Additional file 2: table S1. Pathogenicity prediction of novel variants detected in 28 patients with HSD3B7 deficiency

| Variant  number | Nucleotide change  (NM_025193.4) | Predicted amino acid change (NP_079469.2) | Allele | ACMG classification^†^ | Allele frequency in population | | Prediction | | | | |
| --- | --- | --- | --- | --- | --- | --- | --- | --- | --- | --- | --- |
|  |  |  |  |  | gnomAD | ExAC | PROVEN | Polypen-2 | MutationTaster | SIFT | FATHMM |
| 1 | c.173_174del | p.Val58Glufs*14 | 1 | LP | 0 | 0 | NA | NA | Disease causing | NA | NA |
| 2 | c.319C>T | p.Gln107Ter | 1 | LP | 0 | 0 | NA | NA | Disease causing | NA | NA |
| 3 | c.346T>C | p.Cys116Arg | 1 | VUS | 0 | 0 | Deleterious | Probably Damaging | Disease causing | Damaging | Damaging |
| 4 | c.371T>C | p.Leu124Pro | 1 | VUS | 0.000004 | 0 | Deleterious | Probably Damaging | Disease causing | Damaging | Damaging |
| 5 | c.401G>A | p.Gly134Glu | 1 | VUS | 0 | 0 | Deleterious | Probably Damaging | Disease causing | Damaging | Tolerated |
| 6 | c.402_403insG | p.Pro135Alafs*2 | 1 | LP | 0 | 0 | NA | NA | Disease causing | NA | NA |
| 7 | c.431+2dupT |  | 2 | LP | 0 | 0 | NA | NA | Disease causing | NA | NA |
| 8 | c.484_485delinsCC | p.Ser162Pro | 2 | VUS | 0 | 0 | Deleterious | Probably Damaging | Disease causing | NA | Tolerated |
| 9 | c.485_487delGCA | p.Ser162del | 2 | VUS | 0 | 0 | NA | NA | Disease causing | NA | NA |
| 10 | c.474delC | p.Tyr159Ilefs*27 | 2 | LP | 0 | 0 | NA | NA | Disease causing | NA | NA |
| 11 | c.499G>A | p.Glu167Lys | 2 | VUS | 0.000007 | NA | Deleterious | Probably Damaging | Disease causing | Damaging | Damaging |
| 12 | c.532-3C>G |  | 1 | VUS | 0 | 0 | NA | NA | Disease causing | NA | NA |
| 13 | c.544delC | p.Leu182Cysfs*4 | 2 | LP | 0 | 0 | NA | NA | Disease causing | NA | NA |
| 14 | c.557C>T | p.Thr186Met | 1 | VUS | 0.000024 | 0.000025 | Deleterious | Probably Damaging | Disease causing | Damaging | Damaging |
| 15 | c.561T>G | p.Cys187Trp | 1 | VUS | 0 | 0 | Deleterious | Probably Damaging | Disease causing | Damaging | Damaging |
| 16 | c.569G>A | p.Arg190His | 1 | VUS | 0.000004 | 0.000008 | Deleterious | Probably Damaging | Disease causing | Damaging | Damaging |
| 17 | c.586G>A | p.Gly196Ser | 1 | VUS | 0 | 0 | Deleterious | Probably Damaging | Disease causing | Damaging | Damaging |
| 18 | c.676C>T | p.His226Tyr | 1 | VUS | 0 | 0 | Neutral | Probably Damaging | Disease causing | Damaging | Tolerated |
| 19 | c.682C>T | p.Arg228Trp | 3 | VUS | 0.000008 | 0.000017 | Deleterious | Probably Damaging | Disease causing | Damaging | Tolerated |
| 20 | c.683G>T | p.Arg228Leu | 1 | VUS | 0.000032 | 0 | Deleterious | Probably Damaging | Disease causing | Damaging | Tolerated |
| 21 | c.694+2T>C |  | 1 | LP | 0 | 0 | NA | NA | Disease causing | NA | NA |
| 22 | c.698A>G | p.Asn233Ser | 1 | VUS | 0 | 0 | Deleterious | Probably Damaging | Disease causing | Damaging | Damaging |
| 23 | c.743G>C | p.Arg248Pro | 1 | LP | 0 | 0 | Neutral | Benign | Polymorphism | Tolerated | Damaging |
| 24 | c.770A>G | p.Tyr257Cys | 1 | VUS | 0 | 0 | Deleterious | Probably Damaging | Disease causing | Damaging | Damaging |
| 25 | c.905delA | p.Asn302Metfs*18 | 1 | LP | 0 | 0 | NA | NA | Disease causing | NA | NA |
| 26 | c.920_931delGGCTGCTGCGGC | p.Trp307_Pro311delinsSer | 1 | LP | 0 | 0 | NA | NA | Disease causing | NA | NA |
| 27 | c.964_965dup | p.Leu324Argfs*94 | 1 | LP | 0 | 0 | NA | NA | Disease causing | NA | NA |
| 28 | c.968 C>T | p.Thr323Met | 2 | VUS | 0.000016 | 0.000033 | Neutral | Probably Damaging | Disease causing | Damaging | Damaging |
| 29 | c.968C>G | p.Thr323Arg | 1 | VUS | 0.000016 | 0.000033 | Deleterious | Probably Damaging | Disease causing | Damaging | Damaging |
| 30 | c.988_990delACC | p.Thr330del | 3 | LP | 0 | 0 | Deleterious | NA | Disease causing | NA | NA |
| 31 | c.1033G>T | p.Glu345Ter | 1 | LP | 0 | 0 | NA | NA | Disease causing | NA | NA |
| 32 | c.1040delT | p.Leu347Argfs*70 | 1 | LP | 0 | 0 | NA | NA | Disease causing | NA | NA |
| 33 | c.1061G>C | p.Arg354Pro | 1 | VUS | 0 | 0 | Deleterious | Probably Damaging | Polymorphism | Tolerated | Tolerated |
| 34 | c.-205_323-108del |  | 1 | P | 0 | 0 |  |  |  |  |  |

Abbreviations: NA: not applicable; P, pathogenic; LP, likely pathogenic; VUS, variant of uncertain significance.

† According to the American College of Medical Genetics and Genomics interpretation guidelines.

Additional file 2: table S2. *HSD3B7* (NM_025193.4) variants previously reported in 22 patients with HSD3B7 deficiency

| Variant  number | Nucleotide change  (NM_025193.4) | Predicted amino acid change (NP_079469.2) | Allele | ACMG classification^†^ | **ClinVar ID** | Allele frequency in population | | Reference |
| --- | --- | --- | --- | --- | --- | --- | --- | --- |
|  |  |  |  |  | **(Clinical significance)** | gnomAD | ExAC |  |
| 1 | c.45_46delAG | p.Gly17Leufs*26 | 6 | P | 2885 (P) | 0.000037 | 0.000021 | PMID: 19622360 |
| 2 | c.147G>A | p.Trp49Ter | 1 | LP | NA | 0 | 0 | PMID:31450232 |
| 3 | c.262G>C | p.Gly88Arg | 1 | VUS | NA | 0 | 0 | PMID: 24225297 |
| 4 | c.503G>A | p.Trp168Ter | 9 | P | NA | 0 | 0 | PMID: 26712441 |
| 5 | c.543dupG | p.Leu182Alafs*16 | 6 | P | NA | 0 | 0 | PMID: 26080666 |
| 6 | c.683G>A | p.Arg228Gln | 5 | LP | NA | 0.000032 | 0 | PMID: 26712441 |
| 7 | c.781G>A | p.Asp261Asn | 1 | VUS | NA | 0.000012 | 0.000008 | PMID:31450232 |
| 8 | c.790C>A | p.Pro264Thr | 1 | VUS | NA | 0 | 0 | PMID: 26080666 |
| 9 | c.1031A>G | p.Tyr344Cys | 2 | LP | NA | 0.00002 | 0 | PMID:25946774 |
| 10 | c.1079G>A | p.Trp360Ter | 1 | LP | NA | 0 | 0 | PMID:31450232 |

Abbreviations: NA: not applicable; P, pathogenic; LP, likely pathogenic; VUS, variant of uncertain significance.

† According to the American College of Medical Genetics and Genomics interpretation guidelines.

Additional file 2: table S3. Serum liver biochemistries at first referral and at last follow-up

|  |  | liver biochemistries at first referral | | | | | | | | |  | Liver biochemistries at last follow-up | | | | | | | | | |
| --- | --- | --- | --- | --- | --- | --- | --- | --- | --- | --- | --- | --- | --- | --- | --- | --- | --- | --- | --- | --- | --- |
|  | TB (μmol/L) | DB (μmol/L) | ALT (U/L) | AST (U/L) | GGT (U/L) | TBA (μmol/L) | Alb (g/L) | TCH (μmol/L) | PT  (s) | 25-OH vitamin D3 (ng/ml) | TB (μmol/L) | | DB (μmol/L) | ALT (U/L) | AST (U/L) | GGT (U/L) | TBA (μmol/L) | Alb (g/L) | TCH (μmol/L) | PT  (s) | 25-OH vitamin D3 (ng/ml) |
| P1 | 85.6 | 36.6 | 159 | 154 | 15 | 1 | 49.8 | 3.83 | NA | NA | 5.6 | | 2.4 | 32 | 22 | 17 | NA | 43.5 | 3.38 | NA | NA |
| P2 | 24.7 | 20.1 | 128 | 72 | 8 | 12.4 | 41.8 | NA | 16.3 | NA | 11.4 | | 4 | 13.7 | 23 | 14.7 | 0.7 | 43.3 | NA | 13.8 | 19.1 |
| P3 | 133.9 | 65.5 | 36 | 85 | 29 | 8 | 43.3 | NA | 16.4 | NA | 488.4 | | 343.1 | 268 | 356 | 76 | 111.8 | 34.5 | 2.42 | 44.2 | NA |
| P4 | 137.3 | 102 | 51 | 164 | 33 | 85.4 | 39.6 | NA | 14.8 | NA | NA | | NA | NA | NA | NA | NA | NA | NA | NA | NA |
| P5 | 157.7 | 122.3 | 521 | 356 | 51 | 3.4 | 37.7 | 4.11 | 17.4 | NA | 11.3 | | 4.7 | 5 | 15 | 14 | 2.1 | 43.4 | NA | 14.3 | 25.51 |
| P6 | 96 | 68 | 62 | 46 | 70 | 6 | 34 | NA | 15.2 | NA | 720 | | 593 | 179 | 104 | 155 | 29 | 32 | NA | 17.2 | NA |
| P7 | 123.9 | 75.7 | 157 | 132 | 30 | 2.4 | 41.5 | NA | 43.6 | NA | 260.7 | | 195.5 | 244 | 625 | 19 | 17.4 | 32.1 | NA | 13.4 | NA |
| P8 | 32 | 24 | 51 | 70 | 17 | 1 | 46 | NA | 23.6 | NA | 13 | | 2.6 | 25 | 9 | 10 | 8.2 | 46 | 3.24 | 12.7 | 33.42 |
| P9 | 151.3 | 108.75 | 812 | 819 | 50 | 4.9 | NA | NA | 12.5 | NA | 6.1 | | 2.1 | 16.3 | 25.3 | 16.4 | 2.5 | 45 | 6.08 | 12.8 | 25.33 |
| P10 | 77.4 | 55.1 | 71 | 76 | 21 | 0.4 | 47.1 | 4.31 | 12.2 | NA | 6 | | 2.6 | 10.6 | 27.3 | 12.4 | 4.8 | 42.1 | 3.84 | NA | 27.18 |
| P11 | 164.1 | 109.9 | 376 | 297 | 37 | 23.6 | 42.5 | 4.99 | 13.3 | NA | 163.4 | | 134.5 | 340 | 370 | 96 | 290.7 | 46.1 | NA | 13.9 | NA |
| P12 | 191.4 | 123.1 | 152 | 210 | 37 | 0.2 | 37.3 | NA | NA | NA | 10.6 | | 2.4 | 16 | 31 | 14 | 2.4 | 48.9 | 4.37 | 12 | 53.08 |
| P13 | 103.3 | 85.9 | 284 | 216 | 32 | 8.3 | 40.2 | NA | 13.8 | 12.78 | 12.4 | | 3.7 | 16.8 | 31.6 | 11.2 | 8.8 | 46.1 | 3.97 | NA | 47.75 |
| P14 | 335.9 | 236.8 | 768 | 608 | 29 | 1.3 | 41 | 5.9 | 15.5 | 14.45 | 16.6 | | 2.14 | 13.2 | 24 | 13 | 0.62 | 49.4 | NA | 13.2 | NA |
| P15 | 46.2 | 14.3 | 26 | 34 | 16 | 2 | 44 | NA | 13.6 | 23.39 | 11.5 | | 2.4 | 20 | 25 | 23 | 4.3 | 42 | NA | 12.1 | 46.82 |
| P16 | 98 | 59.3 | 181 | 276 | 28 | 1.2 | 44.9 | 4.98 | 16.3 | 4.8 | 5.1 | | 1.9 | 13.9 | 30.2 | 12.1 | 8.3 | 46 | 4.87 | 12.4 | 40.03 |
| P17 | 81.9 | 37.7 | 75 | 197 | 21 | 1.7 | 45 | 5.09 | 17 | 6.82 | 9.7 | | 3.2 | 25.21 | 40.32 | 14.37 | 11.2 | 43.58 | 4.86 | 13.6 | 24.4 |
| P18 | 82.5 | 51.1 | 83 | 97 | 33 | 6.9 | 45.8 | 3.25 | 15.2 | 3.32 | 7.6 | | 2.8 | 17.48 | 26.43 | 12.2 | 2.5 | 43.95 | 4.49 | 11.3 | 38.08 |
| P19 | 214.7 | 151 | 212 | 282 | 50 | 1 | 37.3 | 4.36 | 16.6 | NA | 3.1 | | 1.7 | 37 | 31 | 14.29 | 5.3 | 42.4 | NA | 12.8 | 39 |
| P20 | 138.1 | 68.8 | 327 | 485 | 29 | 25.4 | NA | 3.84 | 13.5 | 12.77 | 7 | | 2 | 22 | 38 | 27 | 18 | 43 | NA | 12.1 | 22.59 |
| P21 | 309 | 213.6 | 72 | 154 | 50 | 81 | 37.2 | NA | 30.4 | 24.03 | 14.2 | | 4.8 | 42.5 | 48.3 | 46.7 | 124.2 | 41.4 | 3.87 | 14.1 | NA |
| P22 | 20.2 | 13.8 | 47 | 61 | 17 | 1 | 43 | NA | 15.3 | NA | 5 | | 1.9 | 24.53 | 26.98 | 16.83 | 9.8 | 42.39 | 3.83 | 12.7 | 23.53 |
| P23 | 41.4 | 23.3 | 291 | 204 | 26 | 7 | 41.6 | 2.9 | 13.4 | NA | 19.7 | | 10.1 | 151 | 86 | 22 | 0.7 | 47.1 | 2.9 | NA | 7.71 |
| P24 | 141.2 | 70.1 | 134 | 131 | 33 | 1 | 39.5 | 3.61 | 13.8 | 9.82 | 7.9 | | 3 | 31.4 | 43.2 | 12.7 | 4 | 45 | 4.78 | 12.7 | 44.7 |
| P25 | 204.9 | 101.3 | 279 | 393 | 45 | 26.6 | 41.7 | 8.56 | 14.9 | 15.73 | 327.2 | | 150.2 | 116 | 289 | 33 | 80.8 | 32 | NA | 22.8 | NA |
| P26 | 88.9 | 49.5 | 107 | 137 | 33 | 2.6 | 37 | 3.8 | 11.1 | 4.81 | 5 | | 0.8 | 31 | 54 | 264 | 18 | 7.5 | 44 | 12.1 | NA |
| P27 | 125 | 85 | 40 | 132 | 40 | 20.9 | NA | NA | 11.3 | NA | 15.2 | | 5.2 | 41.2 | 44.1 | 10.5 | 2.3 | 42.5 | 4.8 | 13.2 | 42.57 |
| P28 | 165 | 59 | 46 | 294 | 25 | 9.8 | 47 | 3.2 | 14.6 | NA | 201.3 | | 62 | 182 | 662 | 28 | 112.5 | 39 | 3.33 | 19.5 | NA |
| P29 | 96 | 37 | 111 | 167 | 49 | 15.1 | 39 | 4.82 | 12.8 | 27.27 | 6.3 | | 1.5 | 16 | 28 | 17 | 0.5 | 47 | NA | 11.5 | NA |
| P30 | 128.6 | 69.3 | 84 | 406 | 30 | 12.9 | 46 | 4.08 | 15.8 | NA | 126.9 | | 68.6 | 377 | 518 | 31 | 86 | 42 | NA | 12.4 | NA |
| P31 | 15.3 | 3.6 | 40 | NA | 22 | 14 | 38 | 2.81 | 16.1 | 31.87 | 22.8 | | 8.3 | 25 | 33 | 18.4 | 6.1 | 38.2 | 2.5 | 14.7 | 23 |
| P32 | 170.4 | 93.9 | 290 | 153 | 40 | 1.1 | NA | 3.58 | 12.9 | 18.78 | 8.4 | | 5 | 39 | 31 | 45 | 11 | 42.4 | NA | NA | NA |
| P33 | 74.6 | 42.8 | 100 | 200 | 50 | 0.8 | 45 | 3.25 | 14.5 | 18 | 5.5 | | 1.1 | 31 | 49 | 11.9 | 1.6 | NA | NA | 13.5 | NA |
| P34 | 141.2 | 92.1 | 119.8 | 136.7 | 31.8 | 5 | 38.4 | 4.08 | 15.5 | 8.42 | 333.5 | | 273 | 585.1 | 668.1 | 36.5 | 24.4 | 36.9 | NA | 17.1 | 8.5 |
| P35 | 29.4 | 17.9 | 37.6 | 50.2 | 9.9 | 1.2 | 41.6 | 2.57 | 16.6 | 15.69 | 11.8 | | 4.8 | 16 | 24.9 | 14.3 | 4.3 | 44 | 2.73 | 14.7 | 40.24 |
| P36 | 436 | 327.1 | 938.4 | 1526.8 | 65.1 | 4 | 37.6 | 5.21 | 17.6 | 12.09 | 863.1 | | 508.8 | 284.5 | 321.7 | NA | NA | NA | NA | 27.4 | NA |
| P37 | 45 | 35.2 | 217.1 | 385 | 23.1 | 7.7 | 32.2 | 1.54 | 23.2 | 27.04 | 9.1 | | 4.1 | 23.78 | 38.21 | 15.34 | 15.8 | 44.82 | NA | 16.3 | 39.82 |
| P38 | 88 | 66 | 189.3 | 170.5 | 26.5 | 40.9 | 44.5 | 4.22 | 14.2 | 10.08 | 8.2 | | 3.1 | 67.06 | 62.44 | 12.11 | 5.2 | 45.48 | NA | NA | NA |
| P39 | 186.8 | 155.6 | 265 | 428.8 | 28.9 | 1.5 | 44 | 3.94 | 15.7 | 15.5 | 494.5 | | 291.5 | 551 | 559 | 34 | 52 | 25.7 | NA | 25.6 | NA |

Abbreviations: TB, total bilirubin; DB, direct bilirubin; ALT, alanine transaminase; AST, aspartate transaminase; GGT, gamma glutamyl transpeptidase; TBA, total bile acids; Alb, albumin; TCH, total cholesterol; PT, prothrombin time; NA, not available.

Additional file 2: table S4. Correlation of genotype and phenotype in patients with HSD3B7 deficiency

|  | Biallelic  novel variants  (n=17) | Single  novel variant  (n=13) | Biallelic  known variants  (n=9) | Total  (39) | Analysis  (Spearman correlation) |
| --- | --- | --- | --- | --- | --- |
| Group by onset age | | | | | |
| Neonatal cholestasis | 14(82%) | 10(77%) | 8(89%) | 32(82%) | rs=0.038, p=0.817 |
| Childhood onset | 3(18%) | 3(23%) | 1(11%) | 7(18%) |  |
| Clinical Outcome | | | | | |
| Native liver survivors | 11(65%) | 8（62%） | 8（89%） | 27（69%） | rs=0.164, p=0.318 |
| Liver transplanted or death | 6(35%) | 5（38%） | 1（11%） | 12（31%） |  |
